# The association of serum 25-OH vitamin D with asthma in Saudi adults

**DOI:** 10.1097/MD.0000000000012286

**Published:** 2018-09-07

**Authors:** Nasser M. Al-Daghri, Omar S. Al-Attas, Sobhy M. Yakout, Abdullah M. Alnaami, Kaiser Wani, Majed S. Alokail

**Affiliations:** a Biomarkers Research Program; b Prince Mutaib Chair for Biomarkers of Osteoporosis, Biochemistry Department, College of Science, King Saud University, Riyadh, Kingdom of Saudi Arabia.

**Keywords:** Arab adults, asthma, hypovitaminosis D, vitamin D

## Abstract

The study aimed to assess the differences and associations of serum 25 (OH)D levels in Saudi adults with and without asthma. A total of 1070 Saudi adults aged 22 to 28 years (359 with known asthma and 711 matched nonasthmatic controls) were selected randomly from the Riyadh Cohort, Saudi Arabia. Serum 25(OH)D serum levels were measured. Asthma diagnosis was taken from questionnaires. In all participants, 359 (33.6%) were known asthmatic and 711 (66.5%) were nonasthmatic. The overall incidence of vitamin D deficiency (serum 25(OH)D <25 nmol/L) was 29.6% in controls and 35.6% in asthma group (*P* = .01). The asthma group have a significantly lower serum 25(OH)D than the control group (*P* = .01) but lost significance after adjusting for age, body mass index (BMI), and sex. Nonasthmatic and asthmatic females had a higher incidence of vitamin D deficiency (33% and 46%) than nonasthmatic and asthmatic males (17% and 33%). Vitamin D deficiency is significantly high among Saudi adults with asthma, but more so among women. Whether vitamin D deficiency exacerbates asthma attack remains to be proven in this population.

## 1 Introduction

Asthma is a chronic respiratory disease that is widespread worldwide. While it is the most common noncommunicable disease in children, reported deaths are more common in adults.^[1]^ As of 2006, asthma has affected more than three hundred million people worldwide and is responsible for 180 thousand deaths every year.^[2,3]^ Asthma is a common health issue not just in developed but also in developing countries where > 80% of asthma-related deaths occur. The prevalence of bronchial asthma among Saudis as of 2013 was reported to be low at 4.05%.^[4]^

Recently, one of the micronutrients hypothetically involved in asthma etiology has gained significant attention.^[5–7]^ Vitamin D has been considered a strong immune system regulator together with other diseases such as metabolic syndrome, multiple sclerosis, tuberculosis, colorectal cancer, breast cancer, pneumonia, influenza, respiratory distress.^[8]^

Several studies have demonstrated the differences in vitamin D statuses among children with and without asthma.^[9–15]^ Evidence for the link between vitamin D status and asthma is mostly from the pediatric population. Whereas these studies suggest that suitable circulating vitamin D levels are protective with respect to asthma attack exacerbations, the evidence among adults is inconclusive and limited.^[16]^

This present cross-sectional study therefore aims to explore the differences and correlations of serum 25(OH)D among Saudi adults with and without asthma and to observe whether vitamin D status has any influence in determining asthma severity and control among Saudi adults.

## 2 Material and methods

### 2.1 Study subjects

A total of 1070 Saudis adults aged 18 to 50 years old (N = 359 with asthma and N = 711 without asthma) were included. Participants were selected randomly from the Riyadh Cohort as described in details in one of our previous publications.^[17]^ Ethical approval was obtained from the Ethics Committee of the College of Science, King Saud University, Riyadh, Saudi Arabia. Participants were requested to complete the questionnaire which contains demographic information. Clinician-diagnosed asthma was based on the participants’ response to questions: “Has any physician or health professional diagnosed you to have asthma? If yes, what were the medications given to you for this condition and when was the last time you had an attack?” Anthropometry was measured and included height (cm), weight (kg), waist (cm), and hip circumference (cm). Systolic and diastolic blood pressures (mm Hg) were also measured. Body mass index (BMI) was calculated as weight (kg) divided by height (cm) in squared meters.

### 2.2 Biochemical parameters

Fasting blood samples were drawn, centrifuged and serum were placed in plain polystyrene tubes on the same day at primary health centers. Serum samples were sent to the laboratory at King Saud University, Riyadh, Saudi Arabia for storage at −20°C. Fasting serum glucose, total cholesterol, triglycerides, high density lipoprotein (HDL-) and low density lipoprotein (LDL)-cholesterol were measured by routine chemical analyzer (Konelab, Espoo, Finland). Total serum 25(OH)D analysis was performed using commercial electrochemiluminescence immunoassay (Roche Diagnostics, Basel, Germany). This test has an intra- and interassay coefficients of variation of 4.6% and 5.3%, respectively. Vitamin D deficiency was defined according to the recommendations of the American Society for Bone and Mineral Research (ASBMR) ^[18,19]^ with reference ranges defined as follows: deficiency: <25 nmol/L, insufficiency: 25 to 75 nmol/L, sufficiency: above 75 nmol/L.

### 2.3 Statistical analysis

All data in this study were represented as mean ± standard deviation. Median and interquartile ranges were used to represent non-Gaussian variables. Control and asthma groups were compared using *t* test. *P* values<.05 were considered statistically significant. Pearson correlation test was performed to examine various correlations. Analyses were performed using SPSS version 16.0 (SPSS Inc, Chicago, IL).

## 3 Results

### 3.1 Characteristics of the study population

The general characteristics of both groups are presented in Table 1. The incidence of self-reported asthma was 33.5% (N = 359). The control group was significantly older than the asthma group (*P* < .001). Furthermore, the control group had a significantly higher systolic blood pressure, triglycerides, and total cholesterol than the asthma group (*P* values .01,.01, and <.001, respectively). The asthma group on the other hand had a significantly lower level of serum 25(OH)D than the control group, but this significance was lost after adjusting for age, BMI, and sex (*P* = .15). The rest of the measured variables were not significantly different between groups (Table 1).

**Table 1:**
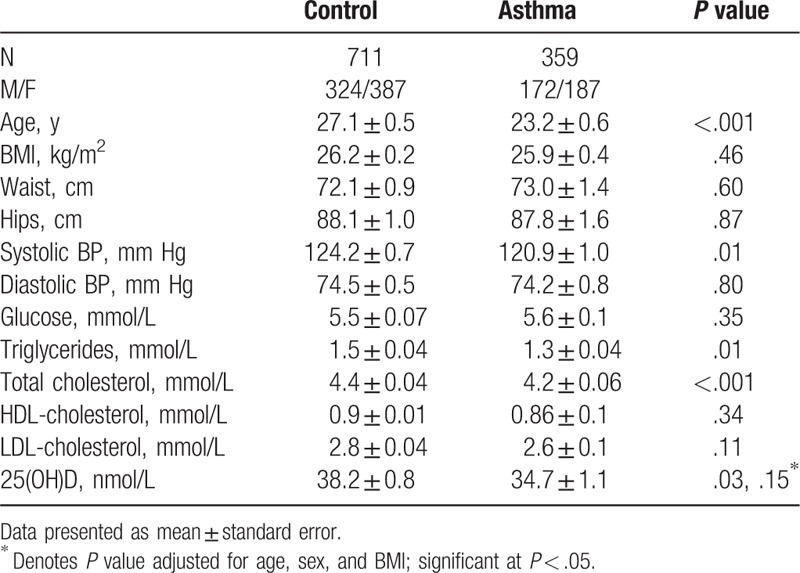
General characteristics all subjects.

### 3.2 Distribution and predictors of 25(OH)D in the study population

**Figure 1:**
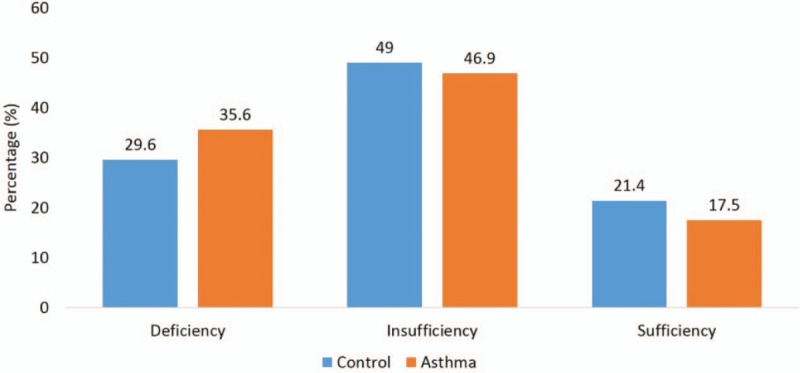
Vitamin D status of control and asthma groups; *P* = .10.

Figure 1 shows the serum 25(OH)D distribution in both groups. The mean 25(OH) D in our study population was 35.9 ng/mL (SD = 8.9). Vitamin D sufficient subjects were found in 21.4% of nonasthmatic participants, in comparison with only 17.5% in the asthmatic group. Vitamin D insufficiency was found in 49% and 46.9% in control and asthmatic groups, respectively. Vitamin D deficiency was observed in 29.6% and 35.6% in control and asthmatic groups, respectively.

**Figure 2:**
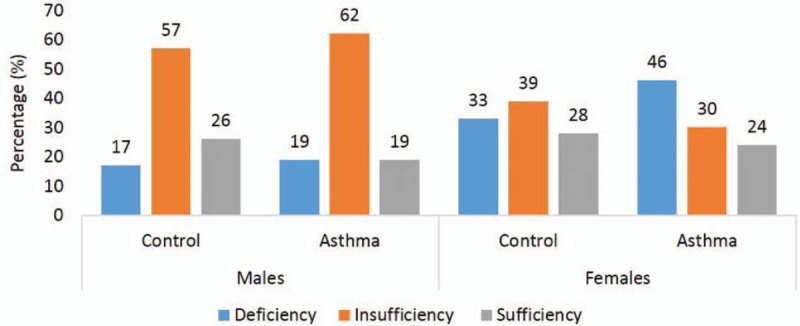
A, Mean differences in 25(OH) vitamin D in adult asthma and control subjects (*P* = NS). B, Percentages (%) in vitamin D status in adult asthma and control groups.

Vitamin D status according to sex between groups was shown in Fig. 2. Control females had a higher prevalence of vitamin D deficiency (33.0%) compared with control males (17%). Furthermore, females in the asthma group had a higher prevalence of vitamin D deficiency (46%) than their male counterparts in the asthma group (33.0%).

### 3.3 25(OH)D levels and asthma

Table 2 shows the bivariate associations of 25(OH)D according to the presence or absence of asthma. In the control, nonasthmatic group, 25(OH)D was significantly associated with age (R = 0.17; *P* < 0.001), glucose (R = 0.07; *P* < 0.05), total cholesterol (R = 0.13; *P* < 0.001), and LDL-cholesterol (R = 0.12; *P* < 0.001). In the asthma group, circulating levels of 25(OH)D was modestly but significantly associated with age (R = 0.12; *P* < 0.05) and HDL-cholesterol (R = 0.11; *P* < 0.05).

**Table 2:**
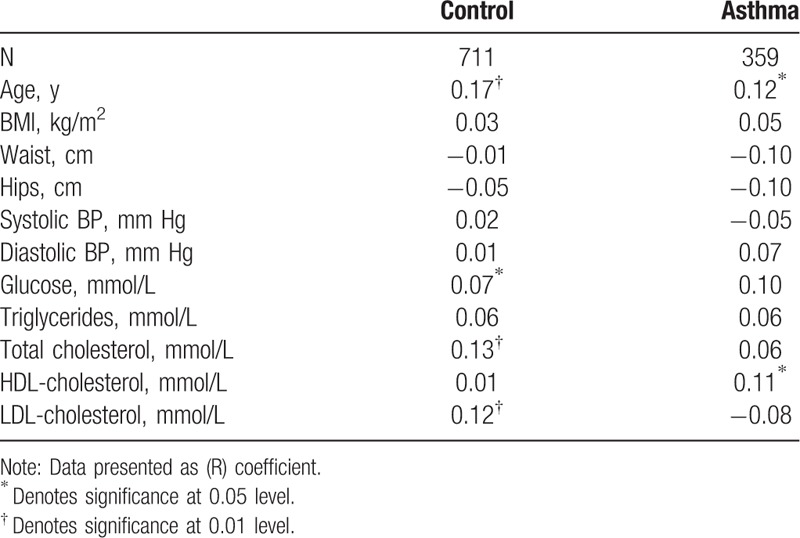
Correlations of log 25(OH) vitamin D with all subjects.

## 4 Discussion

The present study aimed to determine differences and associations of serum 25(OH)D to metabolic parameters among Saudi adults with and without asthma. We found out that 33% and 46% of nonasthmatic and asthmatic women, respectively, had vitamin D deficiency in comparison with only 17% and 19% of nonasthmatic and asthmatic males. Other studies from Saudi Arabia reported around 80% incidence vitamin D deficiency in 1172 women.^[20]^ A number of factors have been implicated for the low vitamin D status in Saudi women including dietary behaviors, absence of exposure to sun, classic clothing,^[3,21]^ darker-skinned, pregnancy, long lactation without vitamin D supplementation,^[22,23]^ limited outside activities, obesity, and absence of government guidelines for food vitamin D fortification. Furthermore, vitamin D-fortified food products in the country are lesser than the suggested regulations by the United States Food and Drug Administration (USFDA).^[24]^

The high incidence of vitamin D deficiency among Saudi women which is most likely due to insufficient exposure to sunlight can be attributed to clothing customs that cover the entire body (e.g., niqab, hijab). Due to this regional culture and customs prevent Middle East, it was suggested that they should try to increase the amount of time they are partially exposed to sunlight and also vitamin D supplementation to prevent vitamin D deficiency. Since the sun is the main source of vitamin D, people receiving more sunlight will most likely have a lesser risk for asthma as observed in several studies.^[25–27]^

The present investigation is one of the few studies assessing the association of vitamin D status and asthma prevalence in Saudi adults. This study was done in 1070 adults (359 asthmatics and 711 controls) to assess the correlation of vitamin D status with asthma. There was a significant association between vitamin D levels and self-reported asthma cases in unadjusted group means. However, when controlling for age, sex, and BMI, the variance did not reach statistical significance. The lack of significant variance in mean vitamin D level between asthmatic participants and controls are in conflict with the results of some studies from other regions, but are nevertheless similar to others. The studies that do support the protective influence of sufficient vitamin D levels were mostly observational.^[28–30]^ A New Zealand study on birth cohort up to 5 years age found no association between vitamin D level and asthma incidence.^[31]^ No association was also found between vitamin D status and asthma in the National Health and Nutrition Examination Survey (NHANES) III participants,^[32]^ whereas the vitamin D was low in children with asthma than those without asthma in American African children.^[9]^ A causal association between vitamin D status and asthma remains to be further explored.^[5]^

Vitamin D can influence asthma pathophysiology through numerous mechanisms. It acts a critical role in adaptive and innate immunity by activating antimicrobial peptides, such as cathelicidin.^[33,34]^ Cathelicidin is active against extensive range of viruses, bacteria, fungi, and mycobacteria. Cathelicidin deficiency is highly associated with infection and asthma exacerbations.^[34]^ Moreover, vitamin D prevents production of Th1-associated cytokines and interleukins like IL-17, thus induces inflammation reduction and smooth muscle cell proliferation.^[31]^ Furthermore, new studies showed that hypovitaminosis D are linked with increased pro-inflammatory cytokine expression, improving pro-inflammatory influence in asthmatic patients. Vitamin D enhances T cells and IL-10 production, reserve of Th2 responses in addition to airway inflammation and hyper-responsiveness.^[35]^

In our study, the absence of correlation between serum vitamin D levels and asthma could be explained by the participants’ ages as all were adults and most studies showing significant correlation focused mainly on early childhood.^[36,37]^ Sufficient vitamin D levels throughout early childhood inhibit respiratory infections, improve lung development, and consequently reduce asthma risk.^[38]^ This explanation mostly works in early life and so vitamin D status might be less significant to asthma persisting to adulthood. Another possible explanation may be due to the cross-sectional design.

The authors acknowledge several limitations. Several important confounders affecting vitamin D such as sunlight exposure, physical activity, diet, and season were not included in the model as these were not available in the dataset. The cross-sectional design also inhibits the study to prove causality. Lastly, the asthma status cannot be confirmed clinically and at best had to be relied on the accuracy of the participants’ self-administration of the questionnaire.

Nevertheless the study also has some strength. Asthma is often recognized via self-reported studies as opposed to physician diagnosis. Moreover, our study population is unique and homogenous. As such, the present findings may offer insights as to how the association of vitamin D and asthma incidence varies according to ethnicity and geographic location.

## 5 Conclusion

While our findings do not specify that serum vitamin D status impacts adult asthma development, we were able to find a higher incidence of vitamin D deficiency among Saudi adults with known history of asthma, more so among women. People at the most risk for the vitamin D deficiency especially asthmatic ones have to be advised to raise their vitamin D supplements and sun exposure.

## Acknowledgment

The authors thank Syed Danish Hussain for the statistical analyses.

## Author contributions

**Conceptualization:** Nasser Al-Daghri, Omar Al-Attas, Majed Alokail.

**Data curation:** Abdullah Alnaami.

**Formal analysis:** Majed Alokail.

**Funding acquisition:** Nasser Al-Daghri.

**Investigation:** Omar Al-Attas, Abdullah Alnaami, Kaiser Wani.

**Methodology:** Sobhy Yakout, Kaiser Wani.

**Project administration:** Abdullah Alnaami.

**Supervision:** Omar Al-Attas, Sobhy Yakout, Abdullah Alnaami.

**Validation:** Kaiser Wani.

**Writing – original draft:** Sobhy Yakout.

**Writing – review & editing:** Nasser Al-Daghri, Kaiser Wani, Majed Alokail.
